# Nitrogen-Broadening Parameters for Atmospheric Spectra Modelling of the ν_3_ Band of SF_6_

**DOI:** 10.3390/molecules27030646

**Published:** 2022-01-19

**Authors:** Nawel Dridi, Vincent Boudon, Mbaye Faye, Laurent Manceron

**Affiliations:** 1 Laboratoire de Spectroscopie et Dynamique Moléculaire, Ecole Nationale Supérieure d’Ingénieurs de Tunis, Université de Tunis, 5 Av. Taha Hussein, Tunis 1008, Tunisia; dridin14@gmail.com; 2 GSMA, UMR CNRS 7331, Université de Reims Champagne Ardenne, Moulin de la Housse B.P039, CEDEX 2, F-51687 Reims, France; 3 Laboratoire Interdisciplinaire Carnot de Bourgogne, UMR 6303 CNRS, Université Bourgogne Franche-Comté, 9 Av. A. Savary, BP 47870, CEDEX, 21078 Dijon, France; 4 Synchrotron SOLEIL AILES Beamline, B.P.48, F-91192 Gif-sur-Yvette, France; mbayefaye516@gmail.com; 5 CNRS, MONARIS, UMR 8233, Sorbonne Université, 4 Place Jussieu, F-75005 Paris, France

**Keywords:** SF_6_, IR absorption, nitrogen-broadening, cross-sections

## Abstract

The infrared absorption of the ν_3_ band region of SF_6,_ at temperatures spanning the 130 to 297 K range, has been reexamined using improved instrumentation with one goal: to estimate the broadening of parameters by nitrogen gas. These parameters are compared to previous literature predictions and an extended set of IR cross-sections is proposed and compared to other existing datasets.

## 1. Introduction

SF_6_, as a synthetic gas with several technological applications, is now present in the atmosphere and, although at relatively small concentration, it has been steadily growing [1,2]. With a long lifetime and very strong absorptions in mid-infrared atmospheric transmission windows, it is an important greenhouse gas to be monitored and one of the species to be controlled mentioned in the Kyoto protocol, due to its exceptionally high global warming power (23,900 times that of CO_2_). Additionally, as it is mostly produced in the northern hemisphere, it is used as a probe to investigate the stratospheric circulation processes and test the currently accepted models; see for instance [3] and references therein. The infrared spectrum of gaseous SF_6_ has been extensively studied at low resolution (see Ref. [4] and references therein) and all its IR-active [5,6,7], Raman active [8,9,10] as well as its IR and Raman-silent [11] fundamentals have been observed at high to very high resolution and analyzed in detail [12]. In particular, the region of the very strongly absorbing ν_3_ mode, which is easiest to detect in situ, is used in various atmospheric spectrometric monitoring experiments [13,14]. Until recently, there existed no line list providing a complete description of the ν_3_ region, as it is cluttered by many hot bands that contribute significantly to the absorption (only about 30% of the population in the ground state near 300 K). The attempts to complete this linelist by adding linelists sequentially provided by the analysis of different transitions involved in the series of (ν_3_ + (*n* + 1)ν_x_) − *n*ν_x_ hot bands [15,16] still fails in the absence of a correct parameter set describing the main hot bands of the (ν_3_ + (*n* + 1)ν_6_) − *n*ν_6_ series. Instead, several authors have proposed empirical cross-sections [17,18] to help quantification in the remote sensing atmospheric spectra, providing spectra at given pressures and temperatures.

However, this useful approach reaches its limits for the correct retrieval of the molecular column in low-pressure stratospheric conditions as spectra cannot be recalculated in the exact physical conditions. Another, innovative approach has been only recently implemented by the Reims and Tomsk groups, which calculated from first principles potential energy and dipole moment multi-dimensional surfaces and used them to compute synthetic spectra, potentially including transitions originating from all populated vibrational levels [19]. This is extremely promising, yet also very challenging in computational power for such large molecular systems with many vibrational degrees of freedom. In [19], Nikitin et al. have thus operated empirical corrections to compensate for the lack of accuracy of the pure ab initio calculations. It thus seems interesting to provide high-quality, high-resolution reference spectra, as well as the most complete linelist possible based on effective Hamiltonians, to serve as reference points for some of the most important transitions. Additionally, even supposing that these theoretical efforts will eventually reach perfection, a good knowledge of the pressure-broadening coefficients and their temperature dependence will still be necessary for the comparison with high-altitude limb atmospheric spectra, where SF_6_ is clearly observable [13,14]. To our knowledge, two studies have been devoted to the assessment of the pressure-broadening coefficients and their temperature dependence. First, the computational study of Tejwani and Fox [20] has calculated the broadening coefficients of SF_6_ by several gases (He, Ar, O_2_, N_2_, HF and SF_6_ itself) at 100, 200, and 300 K within the Anderson–Tsao–Curnutte model. For N_2_, they found a very weak *J*-dependence of the pressure-broadening and calculated values for the γ_0_ coefficient and *n* temperature law exponent of 0.05 cm^−1^/atm on average and 0.623, respectively. More recently, Gamache and coworkers [21] have calculated N_2_-induced pressure-broadening coefficients using the more advanced, complex Robert–Bonamy (CRB) formalism [22] at 200, 250, 296, and 350 K. They also compared their predictions with experimental measurements at 295 K and N_2_ pressures ranging from 200 to 1000 mbar. Their calculations also predict a very weak *J*-dependence of the broadening coefficient. Their estimation of the γ_0_ coefficient and *n* temperature law exponent are 0.078 cm^−1^/atm and 1.05 on average, respectively. In the course of previous studies in our laboratory [23,24,25], we found evidence that the N_2_ pressure-broadening coefficient or its temperature dependence departed noticeably from these predictions and this motivated this new investigation using extended experimental capabilities.

This manuscript is organized as follows: in the first part, new data concerning the ν_3_ region of SF_6_ recorded at improved resolution over a large temperature range and with varied nitrogen pressures are presented. In the second part, we show that the traditional line-by-line retrieval methods are not tractable in such a case and an effective, global approach is presented. From this, an average value of the N_2_-broadening coefficient is determined, even and foremost for a temperature low enough so that the contribution of the ν_3_ fundamental can be clearly separated from that of the hot bands.

## 2. Experimental

Because of the very high IR intensity of the ν_3_ band, a short optical path length at low to room temperature was required. High-resolution spectra were recorded on the Bruker 125HR interferometer AILES Beamline at Synchrotron SOLEIL, using a globar source with appropriate iris aperture diameters, a KBr/Ge beamsplitter and a high-sensitivity home-made, 4 K-cooled HgCdTe detector with a cold 880–1125 cm^−1^ bandpass filter, aperture and focusing optics [26]. Given the strong intensity of the transition, we used the newly developed cryogenic very short path cell regulated at temperature between 130 and 296 K. This setup has been fully described elsewhere [27]. Briefly, it consists of a short, 51 mm long double-walled stainless steel cell with diamond windows, placed in the spectrometer light path under high vacuum (4 × 10^−5^ mbar). Cold gas from liquid-N_2_ evaporation is flowed in the double jacket surrounding the cell to vary the gas temperature. Three temperature sensors, placed inside and outside the cell, are used for temperature measurement and regulation, as well as a resistive heater. Because of the non-linear nature of the feedback loop for the temperature regulation, we estimate a +/−2 K temperature uncertainty only for the 130 to 270 K temperature measurements, +/−1 K for the room-temperature measurements. A high-precision MKS capacitive thermally regulated gauge (1000 mbar range, 0.1% precision) is connected to the cell filling tube to read out the pressure some 30 cm away from the gas cell. Because of the thermomolecular effect [28,29], this can introduce a potential bias in the pressure reading at low temperatures if the gas cell pressure is kept low (less than 1 mbar). To alleviate this problem and for other reasons detailed below, it was decided to keep the total gas pressure larger than 3 mbar. Given the large IR intensity of the ν_3_ fundamental, this meant preparing very dilute SF_6_–N_2_ gas mixtures in a large, well outgassed 3 l stainless steel chamber using 1 mb (for SF_6_) and 1000 mb (for N_2_) Pfeiffer capacitive gauges, and waiting several hours for complete equilibration. Next, precisely measurable pressures of the gas mixture were introduced in the cell. Six series of spectra, corresponding to six different temperatures (130, 169, 213, 230, 270 and 296 K) were recorded at varying SF_6_/N_2_ volume mixing ratios. Spectra were ratioed against an empty cell background, post-zero-filled, corrected for channeling effects and calibrated using well-known water and carbon dioxide rotational lines [30]. The experimental conditions are reported in Table 1.

## 3. Methods

The very high line density due to the small rotational constant, the additional splitting due to centrifugal distortion, and the presence of numerous hot bands had previously prevented a detailed and complete, line-by-line analysis of the ν_3_ region (except for the line positions of the cold band itself [6,12]). Thus, our strategy was first to work at a temperature low enough to restrict observation to lines of the fundamental band. We progressively reduced the gas temperature until the onset of condensation inside the cell was reached, i.e., about 130 K in our experimental conditions. This corresponds to a SF_6_ pressure of the order of less than 0.02 mbar. Since the gas pressure is measured with a pressure gauge regulated at 313 K and placed some 30 cm away from the cold gas cell, the precise measurement of a small gas pressure within the cold cell will be affected by thermomolecular effects [26,27]. To circumvent this difficulty, we diluted the SF_6_ gas in nitrogen to introduce in the cell a total gas pressure well above 2 mbars, where the thermomolecular corrections become negligible. By carefully choosing the starting nitrogen pressure and using a spectral resolution high enough, this also had the advantage of slightly broadening the lines to the point that the correction due to interferometer instrumental line shape (ILS) becomes small to negligible. Next, for each temperature, the nitrogen pressure was progressively increased to evaluate more precisely the nitrogen-broadening effect. As a first test, starting with the lowest temperature at which the spectrum is most simplified, the traditional approach of fitting individual lines in the spectrum using a program taking explicitly into account the ILS was attempted (WSpectra) [31]. The linelist present on the SHeCaSDa website [32,33] was used for positions and the measured gas temperature was used for the Gaussian component of the Voigt profile. Adjustment was thus made on the Lorentzian component.

Due to the severe line overlap, the analysis of only about 260 lines (corresponding to degenerate transitions) could be attempted.

**Figure 1:**
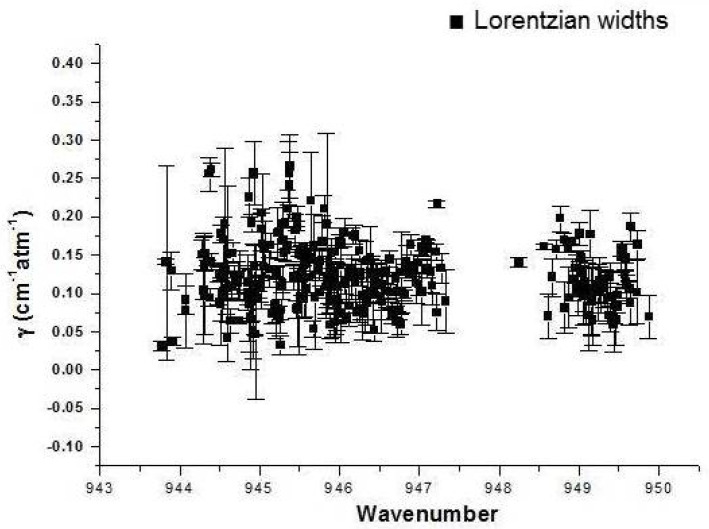
Estimated Lorentzian widths of fitted lines in spectrum P130K 3.27 mb. The error bars are estimations from the fit.

The results are presented in Figure 1 with error bars estimated from the deviation of the fit. It confirms that this approach is too inaccurate to be useful in the case of such dense spectra, but the Lorentzian widths estimated here seem to be close to an average value of 0.125 ± 0.04 (1 σ) and did not seem to indicate a notable dependence in J, confirming on that point the predictions of the theory [20,21]. This motivated us to attempt a more global and effective approach, based on the assumption that the J-dependence of the nitrogen-broadening coefficient and the pressure shifts can be neglected in a first approach. Starting with the coldest spectrum (at 130 K, where the hot bands are very weak), each individual spectrum was recalculated (using the XTDS, SPVIEW [34] and SGEN [35] software) with an effective broadening coefficient. It should be noticed that the XTDS software [34] whose results are reflected in the SHeCaSDa database [32] uses the effective dipole moment derivative for the ν_3_ band that was derived by Person and Krohn a long time ago [36]. This value appears to be reliable, and it did not appear necessary to change it for the present simulations. The very small value of the SF_6_ to N_2_ mixing ratio (0.5% in most cases) justifies neglecting the self-broadening contribution. The program takes into account the correction due to the instrumental and the Doppler contribution.

## 4. Results

**Figure 2:**
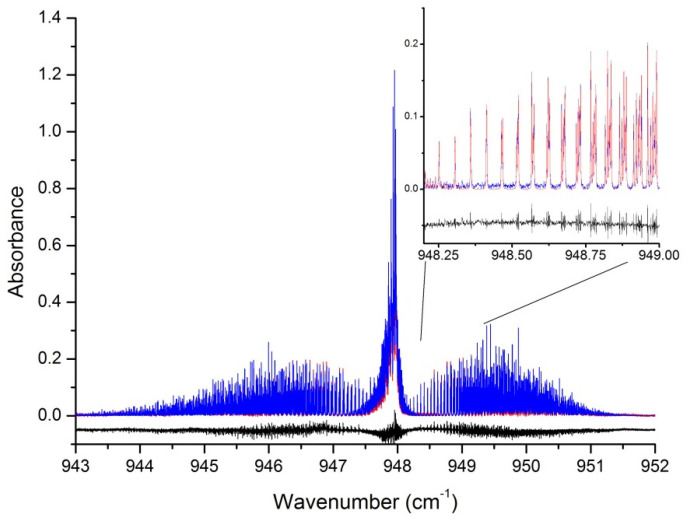
Comparison of experimental spectrum (blue, P130K 3.27mb) and simulated (red) for the ν_3_ region of SF_6_ the temperature is 130(2) K. The black trace is the difference between the upper traces, offset by −0.05 for clarity.

Figure 2 displays the comparison of experimental data and simulation obtained for a value of the broadening coefficient γ(130 K) = 0.131 cm^−1^/atm. The insert presents a detail on the first J clusters on the R-branch side of the band, where lines of the first hot band (ν_3_ + ν_6_ − ν_6_, with a very weak Q-branch near 946.8 cm^−1^) are the least noticeable (at 130 K, the v_6_ = 1 level population is about 7%, while the ground state population represents about 90%).

**Figure 3:**
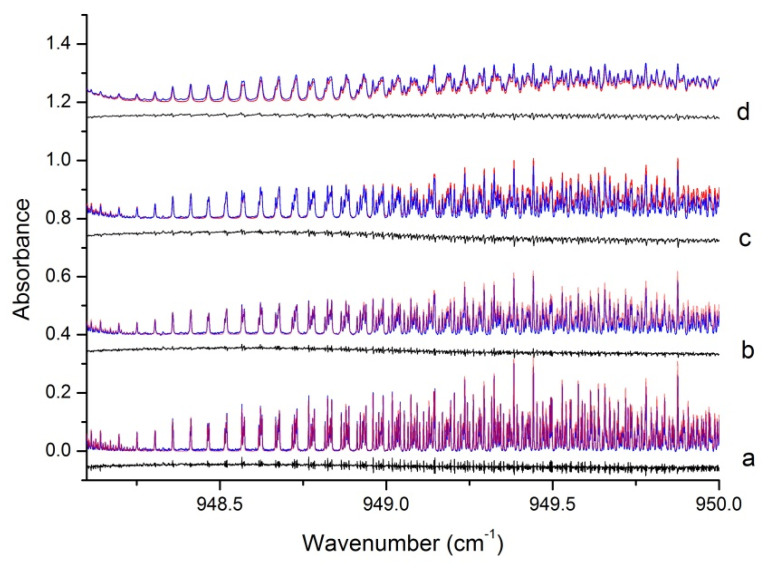
Comparison of experimental (blue), simulated (red, γ = 0.131 cm^−1^/atm) spectra for increasing pressures of nitrogen at 130 K: (**a**) 3.27 mb, (**b**) 6.57 mb, (**c**) 9.46 mb, (**d**) 20 mb. The black traces represent the residuals for each pressure, shifted by −0.05 for clarity.

Please note that the residuals pictured on the black lower trace presents a root mean square deviation of 0.0065 over the whole band (of about 0.25 integrated intensity here), thus about four times that due to the measurement noise. Taking the strongest R cluster lines around 948.7 cm^−1^, the rms value of the difference between observed spectrum and simulation represents 2.6% of the line intensity. Additionally, a departure of 0.02 cm^−1^/atm produces a residual over the whole band that is four times higher. The uncertainty in the broadening coefficient estimate is thus determined, but is as a result of trial-and-error iteration. It is clear that the method does not allow the demonstration of fine differences in the broadening coefficients of lower and higher J lines, but these seem small enough to be within the estimated error bar. Figure 3 presents a comparison for a gradually increasing pressure of nitrogen for the same, low temperature.

**Figure 4:**
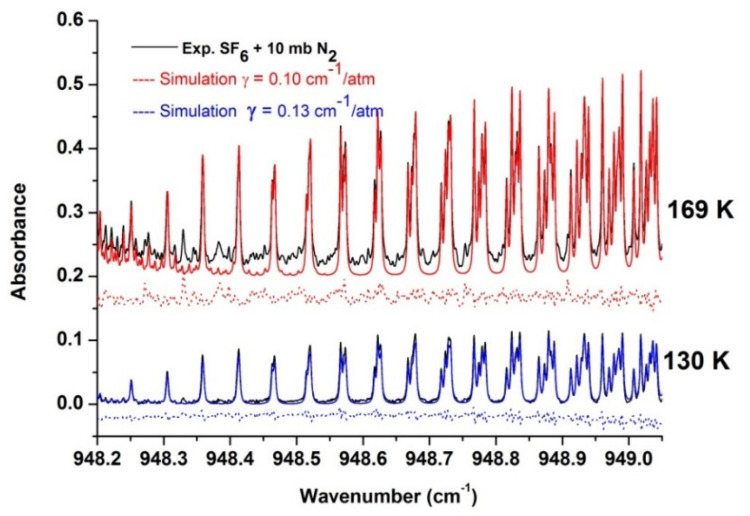
Comparison of experimental (black) and simulated spectra for two temperatures with 10 mb pressure of nitrogen. The dotted lines represent the residuals for each temperature, shifted by −0.05 for clarity.

As the gas temperature rises, the effective broadening parameter must be progressively readjusted. This is somewhat complicated by the hot bands, as hot band line intensities increase progressively. Nevertheless, Figure 4 shows that the simulations remain convincing as the residuals for lines of the fundamental band are well within the hot band lines.

**Figure 5:**
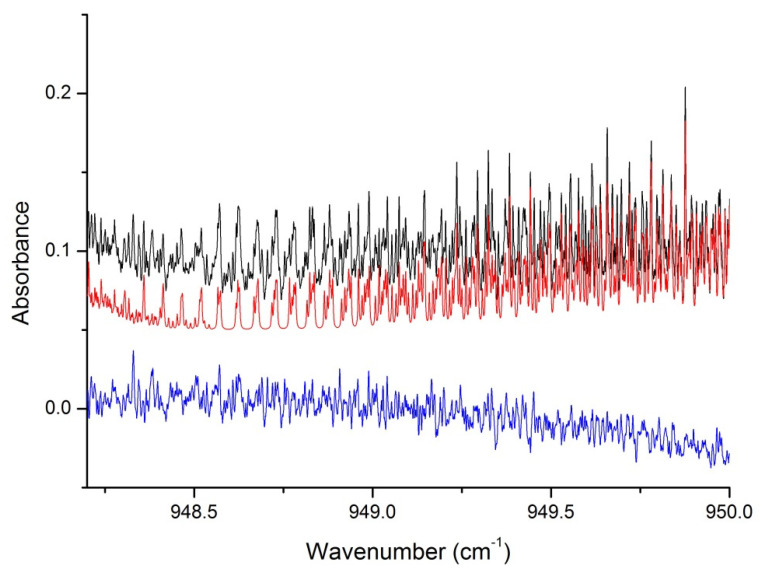
Comparison of experimental (black) and simulated spectra (red) for 297 K with 20 mb pressure of nitrogen. The blue line represents the residuals, shifted by −0.05 a.u. for clarity.

The uncertainty on the estimation of the broadening coefficient increases, however, steadily with the temperature, as the hot band lines gain in intensity. Figure 5 presents a spanning larger view of the simulation in the R-branch at the highest temperature explored in this study.

The estimation of the uncertainty on the effective broadening coefficient takes into account the relative uncertainties on pressure and temperature and, more important, the uncertainty on the adjustment of the observed profile obtained by varying slightly the value of the broadening coefficient around the optimum value until the residuals are clearly above the intensity of the hot band lines. Table 2 presents the estimation for the values of the effective nitrogen-broadening parameter for each temperature along with the estimated uncertainties.

**Figure 6:**
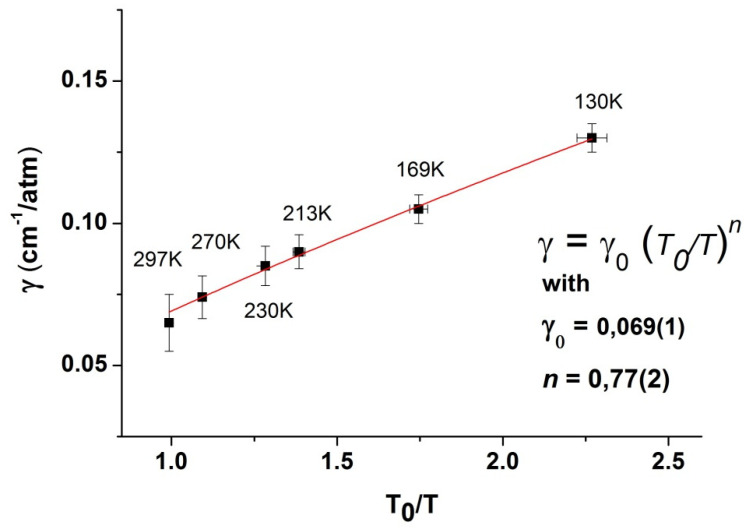
Variation with temperature of the estimated effective nitrogen-broadening coefficient. The red line corresponds to a weighted lest-square fit.

The temperature dependency of the effective broadening coefficient can be modelled using the usual power law:

(1) $$\gamma (T)=\gamma_{{}^{\circ}}\;(T_{0}/T{)}^{n}$$

where the dependency is contained in the exponent *n* and *T*_0_ = 297 K. Figure 6 presents the results along with a linear fit using this power law. Please note that a simple least-square fit results in a convergence towards values close to 0.068 and 0.81 for γ_0_ and *n*, but, when the results are weighted by the uncertainties, somewhat different values are obtained, 0.069 (1) and 0.77 (2) for γ_0_ and *n*. The uncertainties given here correspond to 1 σ deviation from the fit and should be regarded as a lower limit. Given the approximation used in the method, we think it is safer to assume a 3 σ deviation and the values obtained are thus γ_0_ = 0.069 (3) and *n* = 0.77 (6).

Another way to estimate the range of parameters compatible with our results is to take the two straight lines passing through all the error bars with either the higher or lower slopes. This yields the same average values but with larger uncertainties (γ_0_ = 0.069 (5) and *n* = 0.77 (15)).

**Figure 7:**
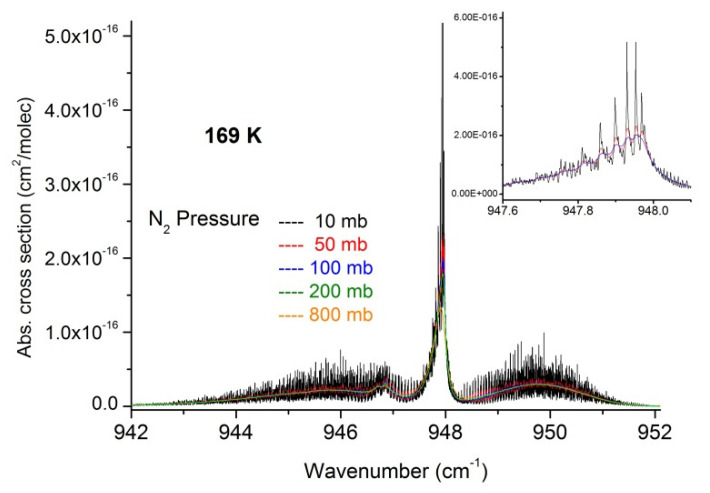
Absorption cross-sections in the ν_3_ region of SF_6_ at 169 K for various nitrogen pressures. The insert shows detail of the Q-Branch for the first three pressures.

**Figure 8:**
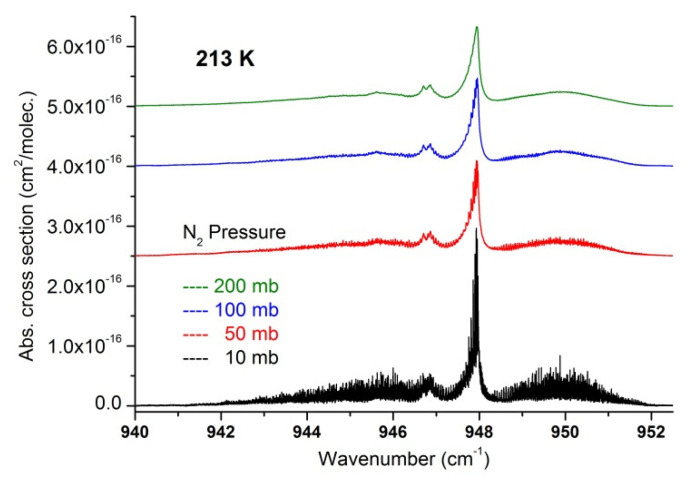
Absorption cross-sections in the ν_3_ region of SF_6_ at 213 K for various nitrogen pressures. The upper traces are offset by steps of 2.10^−16^ for clarity.

**Figure 9:**
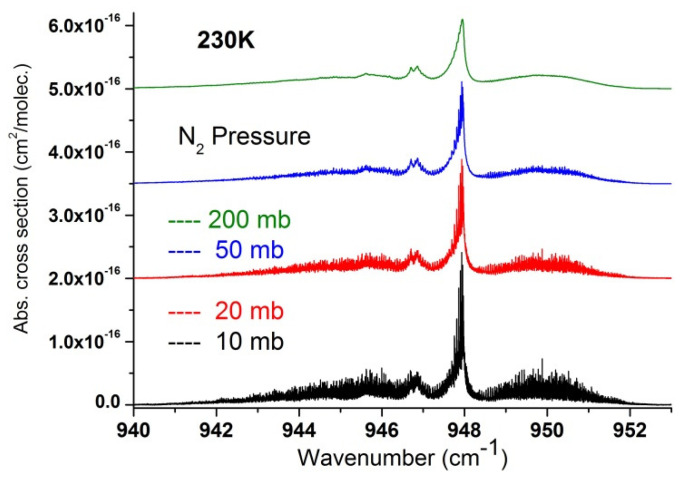
Absorption cross-sections in the ν_3_ region of SF_6_ at 230 K for various nitrogen pressures. Curves for 20 to 200 mb nitrogen pressures are offset for clarity.

**Figure 10:**
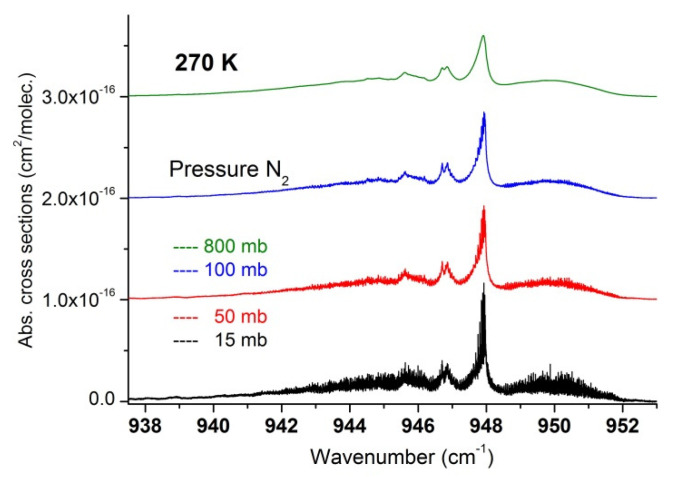
Region of SF_6_ at 270 K for various nitrogen pressures.

**Figure 11:**
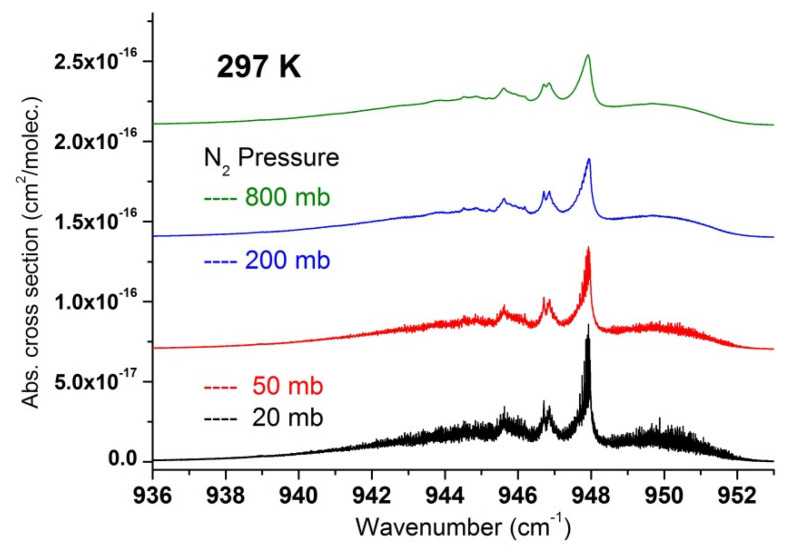
Region of SF_6_ at 297 K for various nitrogen pressures.

In a second step, we used the recorded data to extract a set of absorption cross-sections in temperature and pressure ranges relevant for atmospheric measurements and which may be useful to compare and complete cross-section datasets recently published [17]. These are summarized in Figure 7, Figure 8, Figure 9, Figure 10 and Figure 11 and available in the Supplementary Materials, which can be downloaded on the journal website.

## 5. Discussion and Conclusions

This work represents the first experimental estimation of an average, effective nitrogen-broadening coefficient of the ν_3_ region of SF_6_ and its temperature dependence. The values found here should be compared to the previous theoretical and experimental estimates. The calculation of Tejwani and Fox [20] indicate a very small variation of the broadening parameters over a large range of *J* quantum number (γ (100 K) between 0.108 and 0.097 cm^−1^/atm and γ (300 K) between 0.054 and 0.051 cm^−1^/atm). Their estimate of an average value of γ_0_ is 0.051 cm^−1^/atm and their estimate of the *n* exponent is 0.623. The calculation by Gamache and coworkers [21] also predicted a small variation of γ_0_ with rotational quantum numbers, roughly from 0.074 to 0.0798 cm^−1^/atm and an exponent *n* value around 1.05. [21] also presents a comparison of an experimental spectrum and a simulation based on the γ (295 K) value derived from theory. Their value around 0.075 cm^−1^/atm is close to our estimate (γ_0_ = 0.069(5) cm^−1^/atm), yet slightly outside the error bar of our estimation. Since our estimate accounts quite well for the temperature dependence of the broadening coefficient, we think this value represents at present the best starting point for future simulation of this important spectral region, based on future complete line lists for this region. We want to stress again that the method used here does not allow the demonstration of fine differences in the broadening coefficients of lower and higher J lines, which are likely to exist, but seem small enough to be within the estimated error bar.

The absorption cross-sections presented here are in good general agreement with those presented in the recent publication of Harrison [18], with small differences only. It is important to note that the absorption cross-sections in [18] were normalized to the PNNL integrated absorption intensities of [37], while the present measurements provide an independent determination. The integrated band strength found here for 169, 213, 230, 270, and 297 K are 1.87 (8), 1.88 (8), 1.88 (8), 1.81 (8), and 1.81 (8) × 10^−16^ cm/molecule, thus 1.85 (8) × 10^−16^ cm/molecule on average. This is to be compared to the value of 1.96 (3) × 10^−16^ cm/molecule of [37]. The difference is small and at the limit of the cumulated error bars, but the small dispersion in our measurements gives us confidence that this difference is physically significative and that our data can slightly improve the precision of concentration retrievals. These data have been incorporated in the 2018 release of the technical note for MIPAS data processing [38].

Finally, the inspection of our data, even at moderate resolution with the effect of pressure-broadening, is interesting for the spectral analysis of the region, for which the most intense hot bands have not yet been analyzed. It is interesting to note that it can be clearly seen on the spectra at 130 and 169 K that the *Q*-branch of the first hot band (ν_3_ + ν_6_ − ν_6_) has an asymmetric, two-maxima structure, presumably due to at least two IR-active sub-components (946.70 and 946.85 cm^−1^). The *Q*-branch of the second hot band (ν_3_ + ν_5_ − ν_5_) produces the broad shoulder with two maxima near 945.91 and 946.16 cm^−1^ [15], while the structure near 945.62 cm^−1^, growing next to temperature, is likely to correspond to the (ν_3_ + ν_4_ − ν_4_) and (ν_3_ + 2ν_6_ − 2ν_6_) hot bands. The next challenge will be to supply reliable linelists for all these bands. Combined with the present nitrogen-broadening coefficient, these will supply the means for efficient atmospheric modelling at any temperature and pressure.

## Figures and Tables

**Table 1:** Experimental conditions for the spectra used in this study.

Temperature (K)	Spectrum	P SF_6_ (mbar)	P N_2_ (mbar)	Iris dia. (mm)	Resolution (cm^−1^)	#Scans
130 (2)	P130K 3.27 mb	0.017	3.25	1.15	0.00102	120
130 (2)	P130K 6.57 mb	0.035	6.53	1.3	0.0015	58
130 (2)	P130K 9.46 mb	0.051	9.41	1.7	0.002	60
130 (2)	P130K 20 mb	0.11	19	2	0.003	60
130 (2)	P130K 30 mb	0.16	29.9	2	0.003	60

169 (2)	P170K 10 mb	0.065	10.06	1.3	0.0015	90
169 (2)	P170K 50 mb	0.065	40.1	1.3	0.005	50
169 (2)	P170K 100 mb	0.065	90	1.3	0.005	50
169 (2)	P170K 200 mb	0.065	202	1.3	0.01	50
169 (2)	P170K 800 mb	0.065	803	1.3	0.02	50

213 (2)	P213K 10 mb	0.064	9.94	1.3	0.0015	94
213 (2)	P213K 50 mb	0.064	49.9	1.3	0.005	100
213 (2)	P213K 100 mb	0.064	89.9	1.3	0.005	100
213 (2)	P213K 200 mb	0.064	199.9	1.7	0.01	100

230 (2)	P230K 10 mb	0.064	9.97	1.3	0.0015	120
230 (2)	P230K 20 mb	0.064	20.2	1.7	0.002	80
230 (2)	P230K 50 mb	0.157	49.9	1.7	0.006	60
230 (2)	P230K 100 mb	0.157	100.1	1.7	0.006	60
230 (2)	P230K 200 mb	0.157	200.1	1.7	0.01	60

270 (2)	P270K 15 mb	0.064	9.97	1.3	0.0015	120
270 (2)	P270K 50 mb	0.160	50.0	1.7	0.006	80
270 (2)	P270K 100 mb	0.320	100.0	1.7	0.006	60
270 (2)	P270K 200 mb	0.320	200.1	1.7	0.006	60
270 (2)	P270K 800 mb	0.320	800	1.7	0.02	60

297 (1)	P297K 20 mb	0.129	20.2	1.3	0.0012	480
297 (1)	P297K 50 mb	0.165	50.4	1.3	0.006	480
297 (1)	P297K 100 mb	0.319	100.2	1.3	0.006	100
297(1)	P297K 100 mb	0.319	100.2	1.3	0.006	100
297 (1)	P297K 200 mb	0.319	216.3	1.3	0.01	100
297 (1)	P297K 800 mb	0.320	802	1.3	0.02	100

**Table 2:** Estimated average nitrogen-broadening parameter for the ν_3_ band of SF_6_. Estimated uncertainties are presented in parenthesis in units of the last digit.

Temperature (K)	γ(*T*)
130 (2)	0.131 (5)
169 (2)	0.105 (5)
213 (2)	0.090 (6)
230 (2)	0.085 (7)
270 (2)	0.074 (8)
297 (1)	0.065 (1)

## Data Availability

The cross-sections data presented in this study can be downloaded from the Supplementary Materials.

## Supplementary Materials

The Supplementary Materials are available online. The absorptions cross sections at 169, 213, 230, 273 and 297 K are available online.
